# Understanding public (mis)understanding of tDCS for enhancement

**DOI:** 10.3389/fnint.2015.00030

**Published:** 2015-04-27

**Authors:** Laura Y. Cabrera, Peter B. Reiner

**Affiliations:** National Core for Neuroethics, University of British ColumbiaVancouver, BC, Canada

**Keywords:** cognitive enhancement, neuroethics, public understanding, transcranial direct current stimulation, brain stimulation, public policy

## Abstract

In order to gain insight into the public’s perspective on using the minimally invasive technique transcranial direct current stimulation (tDCS) as an enhancement tool, we analyzed and compared online comments in key popular press articles from two different periods (pre-commercialization and post-commercialization). The main conclusion drawn from this exploratory investigation is that public perception regarding tDCS has shifted from misunderstanding to cautionary realism. This change in attitude can be explained as moving from a focus on an emergent technology to a focus on its applications, benefits, and risks as the technology becomes more grounded within the public domain. Future governance of tDCS should include the concerns and enthusiasms of the public.

## Introduction

Brain stimulation techniques are emerging as methods of neuroenhancement. Among these techniques, transcranial direct current stimulation (tDCS) is one that is gaining public attention as a potential neuroenhancer. This portable technology, which involves applying weak direct currents to the scalp via saline-soaked sponge electrodes, appears rather safe with medical supervision, reasonably effective across a range of brain functions, and accessible to an interested public. These features have led to its growing implementation in both research and clinical settings, as well as with home users (Dubljević et al., 2014).

Given the impact that home use of tDCS may have for individuals and society, changes in public perceptions warrant careful attention, as these may be consequential. In order to gain insight into the public’s attitudes towards tDCS as an enhancement tool, we used thematic analysis to compare online comments on popular press articles from two different time periods: before and after the introduction of the first widely available commercial product. The main conclusion is that the public’s perception regarding tDCS has shifted from misunderstanding to cautionary realism. This change in attitude suggests that as the technology has become more grounded within the public domain, there has been a shift from a focus on an emergent technology to one on its applications and risk-benefit profile.

## Trends in Public Attitudes Towards tDCS

Information on tDCS is growing substantially (Dubljević et al., 2014). While acknowledging that public opinion formation, patterns, and trends can be analyzed and understood through different paradigms, our primary focus here is on attitudinal and perceptual trends as revealed through online comments (Capstick et al., 2014).

### Methods

We conducted a temporal comparison of online comments addressing the use of tDCS as an enhancement tool. Comments on online articles is not a representative sample of the general population, but are available to large numbers of readers from a range of different backgrounds, who can express their opinions by posting comments online. Thus, online communities “offer a mechanism through which a researcher can gain access to people who share specific interests” (Wright, 2005) and diverse opinions (Faridani et al., 2010). We compared two time periods. For our first time period, the EARLIER PERIOD, we only included articles that were published between August 2007 and May 14, 2013, dates that preceded the first offer of a widely available commercially tDCS product to the general public.^1^ We restricted our search to widely read English-language U.S. and U.K. popular media sources that were accessible to readers without a subscription and which had online reader comments. For our second time period, the LATER PERIOD, we included articles from May 15, 2013 to August 2014.

**Figure 1:**
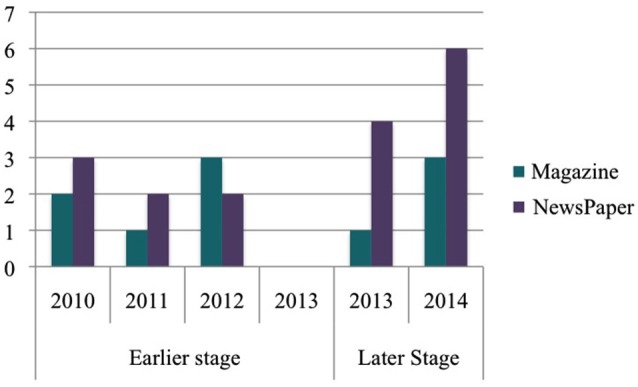
**Distribution of articles according to year published**.

For both time periods, a search was carried out of the Lexis Nexis Academic database and Google using the following terms for newspapers or online magazines: “transcranial stimulation” “tDCS” “neural stimulation” “neurostimulation” “brain stimulation”. The initial search yielded 38 articles for the EARLIER PERIOD and 36 for the LATER PERIOD. Each article was read and checked it for relevance according to pre-established exclusion and inclusion criteria: (1) a focus on tDCS as a cognitive enhancer; and (2) 10 or more comments. The 10 comments inclusion criterion was chosen in order to ensure that the popular media article at hand had generated a good level of discussion. For the EARLIER PERIOD, 13 newspapers (*N* = 8, 61.5%) and magazines (*N* = 5, 38.5%) articles were included for analysis, while for the LATER PERIOD 14 newspapers (*N* = 10, 71.42%) and magazines (*N* = 4, 28.57%) articles were included (see Figure 1).

We employed thematic analysis (Chi, 1997; Braun and Clarke, 2006), with comments coded in an interactive manner, in which themes were developed as the coding process progressed. Themes were grouped into categories. Author replies and comments that were duplicated or irrelevant were excluded, leaving a sample of 248 comments for the EARLIER PERIOD and of 465 for the LATER PERIOD. Inter-rater reliability was determined by randomly selecting 10% of the comments and assigning them to a second coder (Lombard et al., 2002); Cohen’s Kappa was 0.82 for the EARLIER PERIOD and 0.98 for the LATER PERIOD. Descriptive statistics were used to characterize the composition and properties of the sample.

### Limitations

Anonymity of comments can threaten their reliability. Relatively little is known about the demographics of people in online communities (Wright, 2005) which facilitates the posting of polemic, charged and untruthful comments (Lefever et al., 2007). Moreover, we cannot be sure that the posted comment is a result of reading the article or merely responding to the comment thread. Posting of comments is based on volunteer sampling rather than probability sampling (Lefever et al., 2007), and certain websites attract people with like-minded viewpoints, reinforcing biases (Faridani et al., 2010). Finally, our sample composition is limited to English language sources in the United States and United Kingdom, as well as digital natives with access to the Internet. In spite of these limitations, this research provided us with the opportunity to grasp trends and themes regarding the use of tDCS as a cognitive enhancer.

## Results

**Figure 2:**
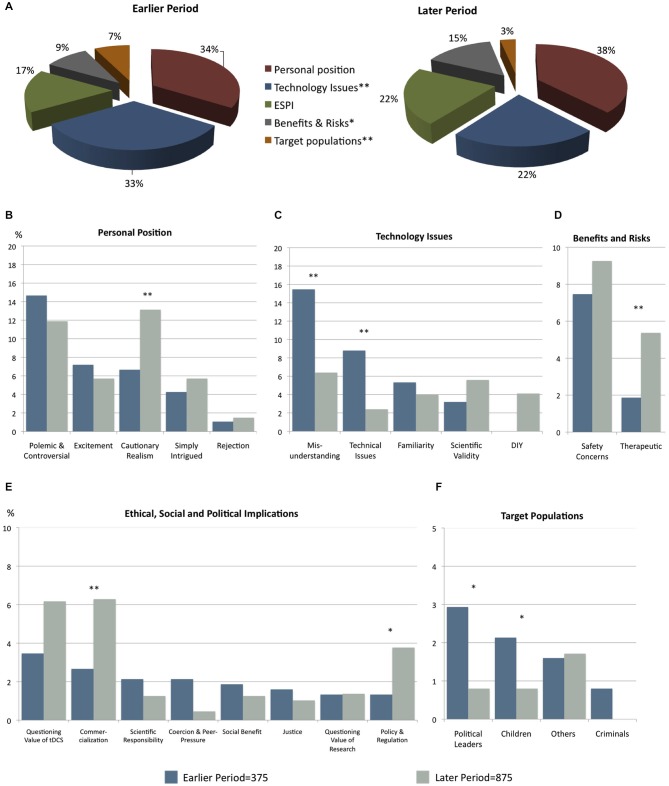
**Comparison of Subjects Addressed within tDCS Online Public Comments**. χ^2^ **p < 0.001 and *p < 0.05. For bar charts *y* axis represents percentage of the overall coded data points. **(A)** Comparison of **categories** earlier and second period. Comparison of **themes** within Category: **(B)** Personal Position **(C)** Technology Issues **(D)** Benefits and Risks **(E)** Ethical, Social and Political Implications **(F)** Target Population.

Personal position and Technology issues were the two most frequent categories of codes for both periods of analysis. Figure 2 displays the comparison between the EARLIER PERIOD and the LATER PERIOD from comments addressing specific categories and themes.

### Early Stage on Public Perception Around tDCS: A Misunderstood Technology

The EARLIER PERIOD was a point in time at which the overall level of understanding of tDCS was limited and there was often conflation with other similar technologies. In many instances, comments addressed tDCS either as an extension of other electricity delivering technologies (such as tasers) or as a form of electroconvulsive therapy (ECT). Technical misinterpretation represented another form of misunderstanding. For instance, there were comments implying unsupported assumptions, such as *the more current or voltage used, the better* the results of cognitive enhancement. There were also comments implying that the current administered by tDCS (generally between 1–2 mA) could lead to “fried brains” or even death.

The other main theme addressed in the comments during the EARLIER PERIOD was their polemic and controversial tone (14.67%, *N* = 55). That enhancement is controversial may have contributed to the controversial tone of many comments, as well as the fact that online comments enable commentators to remain anonymous, creating a space for polemics (Faridani et al., 2010). In addition people’s perceptions are likely to be biased by their hopes, fears, needs and immediate emotional states which can give rise to polarized opinions in pluralistic societies (Pronin et al., 2002).

Even at this early point in time, respondents reported safety concerns in relation to the use of this technology (7.47%, *N* = 28). Ethical issues were not a main category in this sample. For example, even though justice is one of the major concerns regarding cognitive enhancement (Fitz et al., 2014) and tDCS is rather inexpensive compared to other brain stimulation technologies, comments addressing this topic were infrequent. Similarly, comments regarding policy and regulation were also infrequent, despite the fact that a few articles in our sample mentioned the possibility of do-it-yourself (DIY) approaches.

### Second Stage on Public Perception Around tDCS: Cautionary Realism

Whereas the EARLIER PERIOD was marked by a growth in basic awareness and misunderstanding about tDCS, the LATER PERIOD entailed a sustained growth of cautionary concerns overall, a steady polemic and controversial stand, and the proliferation of doubts and skepticism regarding tDCS’s enhancement potential. Comments in this LATER PERIOD focused on subjects about technological based enhancement not being substitution for effort nor the solution for human improvement, the existence of other of non-technological and less risky methods (such as meditation or exercise) for enhancement, and that people can misuse this technology, all captured under the theme cautionary realism.

The overall growth in cautionary concern (*χ*^2^ = 11.07, *p* < 0.001) mirrors a rise in media attention about the use of tDCS as a cognitive enhancer and in particular as a DIY technology. Whereas no single commenter in the EARLIER PERIOD mentioned DIY, this had risen to almost one in ten comments for the LATER PERIOD. Comments mentioning DIY reflected polarized views, as half of the commenters expressed concerns about this practice and the other half enthusiasm.

Skeptical comments centered most prominently on questioning the value of tDCS as an enhancer (*N* = 54) and its scientific validity (*N* = 49). In this LATER PERIOD, comments portraying misunderstanding diminished (*χ*^2^ = 26.03, *p* < 0.001), as expected in a more mature stage of public awareness of the technology.

Compared to the EARLIER PERIOD, comments mentioning technical issues (*χ*^2^ = 26.01, *p* < 0.001) and use of tDCS for political leaders (*χ*^2^ = 8.42, *p* < 0.05) and children (*χ*^2^ = 3.94, *p* < 0.05) were less frequent, whereas comments mentioning commercialization (*χ*^2^ = 6.97, *p* < 0.01), therapeutic benefit (*χ*^2^ = 7.8, *p* < 0.01) and policy and regulation (*χ*^2^ = 5.29, *p* < 0.05) were more frequent. While most comments on policy and regulation reflected concerns about the lack of regulation (*N* = 29), a minority of these explicitly mentioned being against any regulation of tDCS as an enhancer.

## Discussion

### A New Phase for Public Perceptions?

Our results are consistent with other temporal analyses of technology and public understanding, such as those on climate change (Capstick et al., 2014). Before 2012, when the technology was still new in the public sphere, we found widespread misunderstanding of tDCS. In both periods but even more so in the LATER PERIOD, we found that in spite of the loaded and often inadequate representation of tDCS in the media, some commenters distinguished sharply between different brain stimulation techniques and openly criticized the inadequate language and analogies used in the media articles, questioning not only the scientific validity of the articles discussed in the popular media but also the domains to be enhanced.

The availability of tDCS as a consumer device, as well as the vivid online exchange of experiences with tDCS as well as instructions for DIY use (cf.: http://www.reddit.com/r/tDCS/; http://www.diytdcs.com) may be explanatory factors shaping the change in public attitudes towards tDCS, The observation that in the LATER PERIOD misunderstanding was reduced can be regarded as evidence that the public was developing a more mature understanding of tDCS. In view of the past trends, it appears important to inform the public accurately on the short- and long-term consequences of tDCS on healthy individuals and on the plausibility of enhancement effects. In addition, detailed knowledge of the current practice and prevalence of DIY tDCS is also needed.

### Why Public Understanding Around tDCS Matters

Our findings have several implications. As tDCS becomes assimilated into public’s consciousness, beliefs, attitudes, intention and usage of tDCS are likely to change. For example, a flawed understanding of the risk involved could lead to the increased home use. Clear understanding is also of key importance for making informed choices, in this case as a potential consumer of tDCS (Bauer et al., 2006). This becomes a pressing issue if we consider the number of online resources and companies already advertising and promoting a home use of tDCS as a cognitive enhancer. On the other hand, greater familiarity with tDCS and related scientific findings has helped the public to resist pseudo-scientific information, to scrutinize the plausible from the implausible, and to be cautious about using this technology as a cognitive enhancer. Despite the decrease in misunderstanding, the fact that tDCS continues to be confused with ECT may obfuscate the discussion regarding regulation of tDCS. In this view, online comments–ranging from sound counterarguments to the claims made in the article, to personal stories, to seemingly random remarks irrelevant to the article–enable a dynamic construction of meaning and frames in which tDCS can be understood. We invite policy makers to take into account public attitudes and (mis)understanding of tDCS in order to maximize the benefits of innovation while minimizing harms.

## Conclusion

Analyses of comments in online discussion forums are a relevant source to study public attitudes towards tDCS. As this technology continues to mature and more applications become available, researchers have the opportunity to explore trends in public understanding as well as to determine the factors shaping these changes. Our results show that while misunderstanding has decreased as the technology matures, the public seems to become more cautionary and at times skeptical of this technology as a cognitive enhancer tool. From a public policy perspective, analysis of public perceptions over time can help to better inform governance and regulatory frameworks for tDCS.

## Author Contributions

LYC and PBR conception and design of the work, LYC acquisition, analysis and interpretation of data.

## Conflict of Interest Statement

The authors declare that the research was conducted in the absence of any commercial or financial relationships that could be construed as a potential conflict of interest.

## Footnotes

^1^Invitations to pre-purchase a device from foc.us were sent out on that date.
